# A Narrative Review: Repurposing Metformin as a Potential Therapeutic Agent for Oral Cancer

**DOI:** 10.3390/cancers16173017

**Published:** 2024-08-29

**Authors:** Jui-Hsiang Li, Pei-Yi Hsin, Yung-Chia Hsiao, Bo-Jun Chen, Zhi-Yun Zhuang, Chiang-Wen Lee, Wei-Ju Lee, Thi Thuy Tien Vo, Chien-Fu Tseng, Shih-Fen Tseng, I-Ta Lee

**Affiliations:** 1 Division of Endocrinology and Metabolism, Department of Internal Medicine, Taoyuan General Hospital, Ministry of Health and Welfare, Taoyuan 33004, Taiwan; li631001@gmail.com; 2 School of Dentistry, College of Oral Medicine, Taipei Medical University, Taipei 11031, Taiwan; b202112053@tmu.edu.tw (P.-Y.H.); b202112062@tmu.edu.tw (Y.-C.H.); b202112001@tmu.edu.tw (B.-J.C.); b202112077@tmu.edu.tw (Z.-Y.Z.); 3 Department of Nursing, Division of Basic Medical Sciences, Chronic Diseases and Health Promotion Research Center, Chang Gung University of Science and Technology, Chiayi 61363, Taiwan; cwlee@mail.cgust.edu.tw; 4 Department of Respiratory Care, Chang Gung University of Science and Technology, Chiayi 61363, Taiwan; 5 Department of Orthopaedic Surgery, Chang Gung Memorial Hospital, Chiayi 61363, Taiwan; 6 School of Food Safety, College of Nutrition, Taipei Medical University, Taipei 11031, Taiwan; weijulee@tmu.edu.tw; 7 Faculty of Dentistry, Nguyen Tat Thanh University, Ho Chi Minh City 700000, Vietnam; vtttien@ntt.edu.vn; 8 Graduate Institute of Clinical Dentistry, School of Dentistry, National Taiwan University, Taipei 10048, Taiwan; tcf1105@gmail.com; 9 Department of Dentistry, Taoyuan General Hospital, Ministry of Health and Welfare, Taoyuan 33004, Taiwan; 10 Department of Emergency Medicine, Taoyuan General Hospital, Ministry of Health and Welfare, Taoyuan 33004, Taiwan

**Keywords:** metformin, oral squamous cell carcinoma, diabetes mellitus, anticancer properties, epithelial-to-mesenchymal transition, head and neck squamous cell carcinoma

## Abstract

**Simple Summary:**

Oral cancer, particularly oral squamous cell carcinoma (OSCC), is a widespread health problem with limited treatment options and poor survival rates. Metformin, a common medication for managing diabetes, has recently shown promise as a potential treatment for various cancers, including OSCC. This review examines the potential benefits of repurposing metformin to treat oral cancer. Studies have shown that metformin can reduce the growth of cancer cells, induce cancer cell death, and improve the effectiveness of existing treatments. It works by affecting several pathways involved in cancer development, such as those related to cell energy and inflammation. However, using metformin for cancer treatment comes with challenges, including possible side effects and interactions with other medications. More research and clinical trials are needed to understand how metformin can be effectively used in cancer treatment and to ensure its safety. If proven effective, metformin could provide a new, cost-effective option for treating oral cancer, potentially improving outcomes and quality of life for patients.

**Abstract:**

Oral cancer, particularly oral squamous cell carcinoma (OSCC), is a significant global health challenge because of its high incidence and limited treatment options. Major risk factors, including tobacco use, alcohol consumption, and specific microbiota, contribute to the disease’s prevalence. Recently, a compelling association between diabetes mellitus (DM) and oral cancer has been identified, with metformin, a widely used antidiabetic drug, emerging as a potential therapeutic agent across various cancers, including OSCC. This review explores both preclinical and clinical studies to understand the mechanisms by which metformin may exert its anticancer effects, such as inhibiting cancer cell proliferation, inducing apoptosis, and enhancing the efficacy of existing treatments. Preclinical studies demonstrate that metformin modulates crucial metabolic pathways, reduces inflammation, and impacts cellular proliferation, thereby potentially lowering cancer risk and improving patient outcomes. Additionally, metformin’s ability to reverse epithelial-to-mesenchymal transition (EMT), regulate the LIN28/let-7 axis, and its therapeutic role in head and neck squamous cell carcinoma (HNSCC) are examined through experimental models. In clinical contexts, metformin shows promise in enhancing therapeutic outcomes and reducing recurrence rates, although challenges such as drug interactions, complex dosing regimens, and risks such as vitamin B12 deficiency remain. Future research should focus on optimizing metformin’s application, investigating its synergistic effects with other therapies, and conducting rigorous clinical trials to validate its efficacy in OSCC treatment. This dual exploration underscores metformin’s potential to play a transformative role in both diabetes management and cancer care, potentially revolutionizing oral cancer treatment strategies.

## 1. Introduction

Oral cancer, a subtype of head and neck cancer (HNC), encompasses any malignant tissue within the oral cavity. It ranks as the sixth most common cancer worldwide and the second leading cause of cancer-related deaths globally [1]. Among the various types of oral cancer, oral squamous cell carcinoma (OSCC) is the most prevalent, accounting for approximately 90% of cases, and typically develops in the oral lining epithelium. Key risk factors for OSCC include tobacco smoking, alcohol consumption, and certain microorganisms, such as *Fusobacterium nucleatum* (*Fn*) and *Porphyromonas gingivalis* (*P. gingivalis*), as well as viral agents such as human papillomavirus (HPV), which contribute to the disease through various pathogenic pathways [2,3]. Additionally, there is a recognized association between diabetes mellitus (DM) and an increased risk of developing oral cancer, though the mechanisms remain partially understood [4]. 

Interestingly, the antidiabetic drug metformin, commonly used to manage DM, has been identified as having potential protective effects against various types of cancer, including OSCC. The anticancer properties of metformin were first reported in 2005 when its potential was observed in experimental studies [5]. Since then, numerous studies have explored metformin’s effects across different cancer types, including ovarian, breast, colorectal, thyroid, gastric, bladder, and prostate cancers, demonstrating its ability to influence metabolic pathways, reduce inflammation, and inhibit cellular proliferation [6,7,8,9]. These findings suggest that metformin, known for its safety, cost-effectiveness, and favorable profile in treating diabetic patients, may also play a significant role in both the prevention and treatment of cancers [10]. 

A pivotal study in 2020 investigated the link between DM and oral cancer with metformin, involving 500 oral cancer patients and 500 control subjects without precancerous lesions. This study found a negative association between metformin use and oral cancer incidence in DM patients, indicating that metformin users have a lower risk of developing oral cancer [11]. To ensure the quality and relevance of studies reviewed in this paper, we included research published in peer-reviewed journals from 2000 to 2024, specifically focusing on those investigating the effects of metformin on oral cancer and its mechanisms. Our selection encompasses both preclinical studies (in vitro and in vivo) and clinical studies (trials and observational studies) to provide a comprehensive overview. However, studies with incomplete data, unclear methodologies, articles not available in English, and those focused solely on diabetes management without addressing cancer-related outcomes were excluded. 

Preclinical studies have provided insights into the molecular mechanisms by which metformin exerts its anticancer effects. One primary mechanism involves the activation of AMP-activated protein kinase (AMPK), a key regulator of cellular energy homeostasis. By activating AMPK, metformin inhibits the mammalian target of rapamycin (mTOR) pathway, which is critical for cell growth and proliferation, thereby reducing protein synthesis and suppressing cancer cell growth [12]. Additionally, metformin disrupts mitochondrial function by inhibiting complex I of the mitochondrial respiratory chain, leading to decreased ATP production and increased metabolic stress, further activating AMPK and reinforcing the inhibition of mTOR signaling [13]. Metformin also modulates the insulin/IGF-1 signaling pathway, which is frequently overactive in cancer cells, lowering insulin and IGF-1 levels and thereby reducing PI3K/AKT/mTOR pathway activation, which decreases cell growth and induces apoptosis [12]. Moreover, metformin induces cell cycle arrest at the G1 phase by influencing cell cycle regulatory proteins, such as cyclin D1 and p27, promoting apoptosis through pro-apoptotic factors and inhibiting anti-apoptotic proteins [14]. 

Recent research has expanded on these findings, exploring how metformin affects immune cells, the microbiome, and the tumor microenvironment. Metformin has been shown to enhance CD8+ T cell activity while reducing immunosuppressive cells, such as regulatory T cells (Tregs) and myeloid-derived suppressor cells (MDSCs), promoting a stronger anti-tumor immune response [15]. Furthermore, metformin alters the gut microbiome composition, increasing short-chain fatty acid (SCFA) production, which can inhibit histone deacetylases (HDACs) and suppress tumorigenesis [16]. By affecting the tumor microenvironment, metformin reduces glucose and nutrient availability, limiting cancer cell energy supply and making the microenvironment less conducive to tumor growth [17]. These multifaceted effects underscore metformin’s potential as a comprehensive therapeutic agent in cancer treatment, particularly when combined with other therapies targeting these pathways. 

Clinical studies have explored metformin’s potential as a cancer therapeutic agent. While large-scale trials specifically targeting OSCC are limited, extensive research has been conducted on metformin’s efficacy in other cancers, often in combination with established therapies. For instance, a study involving breast cancer patients found that metformin combined with neoadjuvant chemotherapy improved outcomes, indicating its potential as an adjunctive therapy [14]. In another trial, metformin enhanced chemotherapy efficacy in pancreatic cancer patients, suggesting its broader application in cancer treatment [18]. Furthermore, preclinical studies have highlighted metformin’s ability to modulate the tumor microenvironment and enhance immune checkpoint inhibitors’ effects, leading to ongoing investigations into these synergistic effects across various cancers [15]. 

Despite these promising findings, more large-scale, randomized controlled trials specifically focused on OSCC are needed to determine metformin’s efficacy and establish optimized treatment protocols conclusively. Expanding research in this area will not only advance our understanding of metformin’s role in cancer therapy but also enhance its clinical applicability across various cancer types, including oral cancer. Future research should continue to elucidate the detailed pathways through which metformin exerts its anticancer effects and expand clinical trials to establish its efficacy and safety in cancer prevention and treatment. This enhanced understanding highlights metformin’s potential as a multifaceted drug that manages diabetes and offers a promising adjunctive approach in cancer treatment, particularly oral cancer. 

## 2. Classical Application and Mechanism of Action of Metformin

Metformin’s history traces back to *Galega* officinalis, a traditional herbal medicine used in Europe known for its rich guanidine content [19]. Through extensive research, metformin has emerged as a first-line therapy for type 2 diabetes mellitus (T2DM) primarily because of its ability to modulate cellular metabolism [20]. Metformin manages diabetes by enhancing insulin sensitivity and inhibiting gluconeogenesis. As an “insulin sensitizer”, it improves insulin activity and reduces hepatic glucose production by inhibiting gluconeogenesis and decreasing glycogen breakdown. Metformin also indirectly inhibits key enzymes involved in gluconeogenesis and glycogen synthesis in the liver through the activation of AMPK. AMPK plays a crucial role in regulating cellular energy homeostasis; any reduction in liver energy status activates AMPK, which helps restore energy balance [21].

Metformin positively influences blood sugar regulation by enhancing insulin signaling and glucose transport in muscles, distinguishing it from other antidiabetic drugs as it typically does not cause hypoglycemia. Clinical studies have shown that patients treated with metformin or a combination of metformin and glyburide have lower mean fasting plasma glucose concentrations [22]. Metformin’s advantages include its low cost, good tolerability, and mild side effects. However, in rare cases, it can cause lactic acidosis by inhibiting pyruvate carboxylase and gluconeogenesis, leading to lactic acid accumulation [22]. Beyond diabetes management, metformin has been shown to offer cardiovascular and renal protective effects [23]. This is partly because of its role in activating AMPK, which inhibits the mammalian target of rapamycin (mTOR) pathway—a critical regulator of cell proliferation affected by both AMPK and AKT (Protein Kinase B). The mammalian target of rapamycin (mTOR) is a serine/threonine protein kinase in the PI3K-related kinase (PIKK) family, forming the catalytic subunit of two distinct protein complexes, mTOR Complex 1 (mTORC1) and mTOR Complex 2 (mTORC2) [24]. Both complexes are associated with cell proliferation, and the mTOR pathway is influenced by the actions of AMPK and AKT. While AKT activates the mTOR pathway, AMPK inhibits it, suggesting that metformin’s activation of AMPK can suppress abnormal cell proliferation, which is relevant in both diabetes management and cancer prevention [11,25].

Historically, research on metformin focused on its role in managing diabetes symptoms and controlling blood glucose levels [26]. Over time, its potential for treating diabetes-related complications, including nephropathy and cardiovascular diseases, has been explored, often by impacting gene expression, receptor activity, or hormone levels [26]. Increasing evidence suggests that metformin’s ability to influence multiple pathways makes it a promising therapeutic option for diseases beyond diabetes [26]. Given its effects on cellular proliferation, metformin has also been investigated for its potential in cancer therapy. Studies have demonstrated that metformin can inhibit the proliferation of various cancer cells, such as A549 lung cancer cells [27]. Additionally, metformin’s antiviral, anti-inflammatory, and antithrombotic properties have broadened its applications, including as a treatment option for conditions such as SARS-CoV-2 [28].

In summary, the therapeutic potential of metformin extends beyond its primary use as an antidiabetic drug. Its mechanisms of action, including AMPK activation and mTOR pathway inhibition, indicate a promising role in cancer prevention and treatment. Metformin’s impact on various metabolic and inflammatory pathways suggests its utility in addressing a wide range of diseases. Future research should focus on elucidating the detailed mechanisms through which metformin exerts its effects and expanding clinical trials to confirm its efficacy and safety in non-diabetic conditions, including cancer.

## 3. Novel Applications for Metformin

### 3.1. Metformin’s Efficacy on the Cardiovascular System and Kidneys

Metformin’s therapeutic benefits extend beyond diabetes management, encompassing significant cardiovascular and renal protective effects. Clinically, metformin has been shown to reduce cholesterol levels and prevent the formation of atherosclerotic plaques, thereby lowering the risk of coronary artery disease and related complications. Its inhibition of cytokine expression through the AKT/MAPK pathway also underscores its anti-inflammatory properties, which are crucial for preventing cardiovascular events, particularly those triggered by infections such as influenza A virus (IAV) [29]. In addition, metformin upregulates the SLC25A47 gene, essential for lipid metabolism, which helps in the removal of excess LDL cholesterol and protects against hepatic steatosis and subsequent cardiovascular risks [30]. In patients with heart failure, metformin has been observed to reduce the levels of N-terminal pro b-type natriuretic peptide (NT-proBNP), a biomarker associated with heart failure, and limit cardiomyocyte apoptosis. By activating AMPK and enhancing nitric oxide (NO) production, metformin prevents adverse cardiac remodeling and promotes vascular health, thereby improving long-term outcomes for heart failure patients [31,32].

Metformin also shows promise in managing diabetic kidney disease (DKD). It reduces the expression of receptors for advanced glycation end products (AGEs) and inhibits the production of reactive oxygen species (ROS), both of which contribute to kidney inflammation and fibrosis [33]. By mitigating these harmful processes, metformin can significantly slow the progression of DKD, making it a valuable therapeutic option for managing this major complication of T2DM. Overall, metformin’s diverse therapeutic effects extend well beyond glycemic control, providing robust cardiovascular and renal protection. Its ability to modulate various metabolic and inflammatory pathways suggests its potential to treat and prevent a wide range of conditions beyond diabetes. Future research should aim further to elucidate the full spectrum of metformin’s benefits and optimize its application across multiple health domains to maximize patient outcomes.

### 3.2. Metformin’s Efficacy on Polycystic Ovary Syndrome (PCOS)

Studies have shown that combination therapy with metformin and empagliflozin can significantly improve ovarian function in women with polycystic ovary syndrome (PCOS) by upregulating the expression of AMPKα and sirtuin 1 (SIRT1) [34]. PCOS is often characterized by a reduction in mature follicles and corpora lutea, leading to impaired reproductive function, and is frequently associated with metabolic disturbances such as dyslipidemia, which increase the risk of cardiovascular diseases [35,36]. Preclinical studies have demonstrated that the dual therapy of metformin and empagliflozin enhances follicular growth, thickens the granulosa cell layer of follicles, and corrects dyslipidemia, thereby reducing cardiovascular risk factors and supporting overall metabolic health [34,37,38]. In clinical settings, empagliflozin, a sodium-glucose co-transporter-2 (SGLT2) inhibitor, complements metformin’s actions by promoting glucosuria, which helps lower both fasting and postprandial glucose levels [38]. This mechanism improves insulin sensitivity and aids in weight reduction, a particularly valuable benefit for women with PCOS who often struggle with obesity [34,37]. Additionally, the combination therapy exerts anti-inflammatory effects, reducing markers of systemic inflammation commonly elevated in PCOS patients. These anti-inflammatory effects are linked to the activation of AMPKα and SIRT1 pathways, which are crucial in regulating energy homeostasis, inflammation, and oxidative stress, thereby improving mitochondrial function.

Metformin primarily achieves these effects by inhibiting complex I of the mitochondrial respiratory chain, reducing ATP production, and increasing the AMP/ATP ratio, which activates AMPK. This activation enhances mitochondrial biogenesis, optimizes oxidative phosphorylation, and reduces the production of ROS, contributing to better mitochondrial efficiency and overall cellular homeostasis [39]. Recent studies suggest that this therapeutic combination may enhance endothelial function and reduce the risk of cardiovascular events, further supporting its use in managing the long-term complications associated with PCOS [38]. Long-term use of metformin alone has been shown to improve ovulatory function and metabolic parameters in women with PCOS, effectively addressing both hormonal and metabolic imbalances characteristic of the condition [36]. Moreover, the addition of empagliflozin to metformin therapy has demonstrated benefits in managing metabolic parameters in T2DM, suggesting its potential utility in metabolic regulation for PCOS patients as well [40]. Continued research and clinical trials are essential to fully elucidate the long-term benefits and mechanisms of action of this combination therapy in PCOS management. The ability of this therapy to address both reproductive and metabolic dysfunctions makes it a promising option for improving the quality of life in women with PCOS.

### 3.3. Metformin’s Efficacy on Cancer Treatment

Research has demonstrated that individuals with T2DM treated with metformin experience a reduced rate of cancer-related deaths. Metformin exerts its anticancer effects primarily by inhibiting cell proliferation and targeting the mitochondrial respiratory chain complex I [8,41]. By binding to complex I, metformin decreases adenosine triphosphate (ATP) synthesis and increases the ratio of nicotinamide adenine dinucleotide (NADH) to NAD+. This alteration in ATP and NADH levels inhibits gluconeogenesis, leading to decreased insulin-like growth factor 1 (IGF-1), a key factor in cell proliferation and mitogenesis [8].

Extensive research is exploring metformin’s effects across various cancer types, including breast, colorectal, and oral cancers. Preclinical studies have provided significant evidence suggesting that metformin treatment is associated with a reduced risk of breast cancer. In laboratory models, metformin monotherapy inhibits the development of MCF-7 and SKBR-3 breast cancer cell colonies and prevents tumor invasion [42]. When combined with tamoxifen, a commonly used breast cancer drug, metformin can decrease DNA concentration by approximately 65% compared with the control group in the chorioallantoic membrane (CAM) ex ovo model [42]. This finding demonstrates metformin’s potential to enhance the anti-metastatic effects on breast cancer cells in experimental settings.

Preclinical research has also shown that metformin significantly modulates gut microbiome (GMB) composition, which may play a role in its antitumor effects. The modulation of the GMB by metformin is thought to contribute to tumor growth suppression, likely through complex interactions within the tumor microenvironment [16]. Additionally, metformin influences cholesterol synthesis regulation by transcription factors, such as sterol response element-binding protein (SREBP). In experimental models, metformin induces butyrate formation, which can inhibit SREBP and cholesterol synthesis, thereby reducing the potential for cancer progression [43].

In the context of OSCC, preclinical studies indicate that metformin inhibits mTORC1 activity through pathways involving IGF1 and IGF2, contributing to reduced cancer cell proliferation [44]. Metformin acts on oral cancer through the organic cation transporter (OCT3), and studies have identified high levels of OCT3 expression in head and neck squamous cell carcinoma (HNSCC) and oral premalignant lesions (OPLs). Given that OPLs and their stromal environment require time to remodel, prolonged metformin treatment may be necessary to increase response rates [45]. Additionally, preclinical studies suggest that metformin may modulate immune responses by enhancing tumor-infiltrating T-cell function, which can be assessed through immune markers such as CD8, CD163, and PD-L1 [46].

The interaction between metformin and the immune system further underscores its comprehensive approach to cancer treatment in experimental models. By enhancing adaptive immunity, reducing inflammatory responses, and modulating metabolic pathways, metformin offers a multi-faceted strategy for combating cancer. Future research should continue to explore the mechanistic details of metformin’s effects across different cancer types to expand its clinical applications and potentially improve patient outcomes.

In summary, metformin’s multifaceted mechanisms—including inhibition of mitochondrial complex I, modulation of IGF-1, and effects on the gut microbiome—contribute to its anticancer properties in preclinical models. Its ability to inhibit cell proliferation, induce apoptosis and modulate immune responses highlights its potential as a valuable adjunct in cancer therapy. Continued research, including clinical trials, is essential to elucidate the pathways through which metformin exerts its effects fully and to optimize its use in clinical oncology.

## 4. The Therapy for Oral Cancer

Traditional therapies for oral cancer primarily include radiotherapy, chemotherapy, and surgery. These treatments remain the cornerstone of tumor management [47]. However, they come with significant drawbacks. Surgery can cause substantial damage to the oral cavity and face, while radiotherapy often leads to permanent xerostomia and radiation caries. Chemotherapy is frequently associated with high resistance rates and severe systemic adverse effects, making it challenging to completely eradicate tumors in the oral cavity, resulting in an overall 5-year survival rate of approximately 50% [48,49]. Furthermore, the high potential for local infiltration, lymph node metastasis, and delayed diagnosis significantly complicate the effective treatment of oral cancer using conventional methods [47]. To overcome these challenges, researchers are actively exploring new therapeutic approaches and have recently made significant breakthroughs. Innovations include the use of vitamin D, targeted drug therapy, photothermal therapy, nanotechnology, and, notably, metformin [49,50,51]. These advancements offer promising alternatives that could potentially enhance the efficacy of treatment and improve outcomes for patients with oral cancer.

### 4.1. Vitamin D

Vitamin D has been well-documented for its effectiveness in treating various cancers, including breast and digestive tract cancers, as evidenced by recent studies [52,53,54]. Emerging research from preclinical studies indicates that a deficiency in vitamin D significantly increases the risk of developing oral cancer [55]. In laboratory settings, vitamin D has been shown to play a role in regulating inflammation and prostaglandin synthesis and inhibiting metastasis, positioning it as a promising agent for both the prevention and treatment of tumors [56]. Furthermore, vitamin D is essential for the regulation of calcium and phosphorus, which are critical components for maintaining oral health and strengthening teeth [50]. 

Preclinical studies have demonstrated that high-dose oral vitamin D (oral hdVD) can be an effective strategy for eliminating cancer cells [57]. This mode of administration ensures that sufficient levels of vitamin D are available to exert its anticancer effects, including the modulation of the immune system, enhancement of cellular differentiation, and induction of apoptosis in cancer cells [58]. These mechanisms collectively contribute to reducing tumor growth and preventing the spread of cancerous cells in experimental models. Vitamin D’s anti-inflammatory properties are particularly beneficial in the context of cancer prevention and treatment. By reducing chronic inflammation, a known risk factor for various cancers, vitamin D helps create a less favorable environment for cancer development in preclinical studies [59]. Moreover, its role in prostaglandin synthesis regulation helps in controlling cell proliferation and differentiation, further preventing cancerous growth [56]. 

In addition to its potential anticancer properties demonstrated in preclinical studies, vitamin D’s ability to regulate calcium and phosphorus homeostasis is crucial for oral health. Adequate levels of these minerals are necessary for maintaining the structural integrity of teeth and bones, which not only helps in preventing dental caries and other oral health issues but also supports overall oral and systemic health [50]. The potential of vitamin D in cancer therapy is further supported by its ability to enhance the effects of other treatments in experimental settings. For instance, combining vitamin D with conventional therapies such as chemotherapy and radiotherapy has been shown in preclinical models to improve treatment outcomes and reduce side effects [60]. This synergistic effect makes vitamin D a valuable adjunct in comprehensive cancer care. 

In light of these findings from preclinical research, incorporating vitamin D supplementation into standard cancer prevention and treatment protocols could offer significant benefits. However, ongoing clinical research is essential to determine the optimal dosing and administration methods to maximize the therapeutic effects of vitamin D in oral and other cancers. Clinical trials are currently underway to explore its potential and establish evidence-based guidelines for its use in oncology [61]. 

In summary, vitamin D has demonstrated considerable potential in both preventing and treating oral cancer, as well as other types of cancer, particularly in preclinical studies. Its multifaceted roles in inflammation regulation, mineral homeostasis, and synergistic enhancement of conventional therapies underscore its value as a strategic component in cancer management. Future studies and clinical applications will continue to reveal the full scope of vitamin D’s benefits in oncology, offering hope for improved patient outcomes and enhanced quality of life [62].

### 4.2. Target Drugs

Targeted drug therapies are designed to block specific molecules and pathways that are crucial for cancer growth and progression. Researchers have developed advanced drug delivery systems specifically for oral cancer to deliver drugs to or penetrate them precisely into target tissues. This targeted approach aims to attack cancer cells directly while minimizing adverse side effects, thereby reducing toxicity to normal cells and improving patient survival rates [63,64]. 

In preclinical research, one significant target in cancer therapy is the mitochondria because of its critical role in energy generation and cellular metabolism. Drugs targeting mitochondrial functions have shown promise in inducing cancer cell death and overcoming drug resistance in laboratory studies [65]. For example, menadione induces the production of ROS, leading to oxidative stress and apoptosis in cancer cells. Menadione also has the potential to prevent drug resistance, thereby enhancing the efficacy of chemotherapy in experimental models [66,67]. 

Another mitochondrial-targeted drug, pyrvinium pamoate, a complex II inhibitor, has been shown in preclinical studies to reduce tumor sphere formation in cancer cell lines, indicating its potential to hinder cancer stem cell-like properties and metastasis [68]. Moreover, complex III inhibitors such as antimycin A and atovaquone have been found to selectively increase glycolysis rates in cancer cells in vitro [69]. By disrupting the mitochondrial electron transport chain, these inhibitors force cancer cells to rely on glycolysis for energy production, leading to metabolic stress and cell death [69]. This selective targeting of cancer cell metabolism presents a promising strategy for cancer treatment, potentially reducing the side effects associated with traditional therapies. 

Metformin, traditionally used to treat diabetes, has also been explored as a targeted drug in cancer therapy. In preclinical studies, its ability to inhibit mitochondrial complex I and activate AMPK suggests potential mechanisms through which it may exert anticancer effects [10]. However, the precise role of metformin in oral cancer remains under investigation. Some preclinical studies suggest that metformin may interfere with cancer cell metabolism and proliferation, while others propose its potential to enhance the efficacy of other anticancer treatments [14,70]. 

Furthermore, the development of novel drug delivery systems has significantly advanced the field of targeted therapy. In experimental settings, nanoparticles, liposomes, and other nanocarriers are being utilized to improve drug delivery to cancer cells, ensuring higher concentrations of the drug reach the tumor site while sparing healthy tissues [71]. These innovative delivery systems enhance the therapeutic index of anticancer drugs, reduce systemic toxicity, and improve patient outcomes in preclinical models. 

In addition to mitochondrial targeting, researchers are exploring other molecular targets in oral cancer, such as growth factor receptors, signaling pathways, and genetic mutations specific to cancer cells. By designing drugs that specifically interact with these targets, it is possible to inhibit cancer cell growth, induce apoptosis, and prevent metastasis more effectively in laboratory studies [72]. This precision medicine approach holds great promise for the future of cancer therapy, offering personalized treatment strategies based on the molecular profile of each patient’s tumor. 

In conclusion, targeted drug therapies represent a significant advancement in the treatment of oral cancer, as demonstrated in preclinical research. By focusing on specific molecules and pathways involved in cancer progression, these therapies aim to enhance treatment efficacy while minimizing side effects. The ongoing development of advanced drug delivery systems and the exploration of new molecular targets continue to push the boundaries of cancer treatment, offering hope for improved survival rates and better quality of life for patients in the future. Continued research and clinical trials are essential to fully realize the potential of these innovative therapies in the fight against cancer.

### 4.3. Photothermal Therapy

Photothermal therapy employs photothermal agents (PTAs) to convert light energy into heat through the photothermal effect (PTE), leading to the ablation of tumor cells [49]. One commonly used PTA is indocyanine green (ICG), valued for its near-infrared light absorption properties and safety profile, which has been approved by the FDA. However, a limitation of ICG is its compromised accumulation in tumor cells due to the rapid uptake by plasma proteins [73,74]. To address this, liposomal ICG formulations have been developed. These formulations, with their larger size and modified pharmacokinetic profiles, enhance tumor accumulation and retention, making them more effective for photothermal therapy [73].

In the preclinical context of oral cancer treatment, three main types of PTAs are used: metallic compounds, carbon-based materials, and organic materials. Metallic compounds, such as gold nanoparticles, provide excellent photothermal conversion efficiency and biocompatibility, making them highly effective for tumor ablation in laboratory settings [75]. Carbon-based materials, including graphene and carbon nanotubes, are noted for their high thermal conductivity and stability [76]. Organic materials, such as conjugated polymers, offer the advantages of biodegradability and functional versatility [77]. 

While these PTAs show promise in preclinical studies, their limited efficacy often necessitates combination with other therapeutic agents or modification with functional molecules to enhance tumor-killing efficacy [78]. Combining PTAs with other therapeutic modalities, such as chemotherapy, radiotherapy, or immunotherapy, has demonstrated synergistic effects in preclinical models, improving overall therapeutic outcomes [79]. For instance, gold nanoparticles can be functionalized with chemotherapeutic drugs to provide simultaneous photothermal and chemotherapeutic effects, enhancing cancer treatment efficacy in experimental setups [80]. 

Similarly, carbon-based materials can be conjugated with targeting ligands or immune-modulating agents to selectively target tumor cells and modify the tumor microenvironment, boosting the immune response against cancer cells [81]. The integration of nanotechnology in photothermal therapy has led to the development of advanced nanocarriers in laboratory studies. These carriers deliver PTAs directly to the tumor site, minimizing off-target effects and maximizing therapeutic efficacy [82]. Engineered to respond to specific stimuli, such as pH or temperature changes, these nanocarriers ensure the controlled release of PTAs and enhance their accumulation in tumor tissues [83]. 

Despite significant advancements in preclinical research, several challenges remain before photothermal therapy can be widely implemented in clinical practice. These challenges include achieving precise control over the biodistribution and targeting of PTAs, minimizing potential side effects, and ensuring the long-term safety and biocompatibility of the materials used [84]. Additionally, optimizing light delivery systems to achieve uniform and deep tissue penetration is crucial for effective clinical application [85]. 

In conclusion, photothermal therapy represents a promising approach for treating oral cancer, leveraging the unique properties of PTAs to achieve targeted tumor ablation. The ongoing development of advanced PTAs, combined with innovative drug delivery systems and multimodal therapeutic strategies, holds great potential for enhancing the efficacy and safety of photothermal therapy. Continued research and clinical trials are essential to overcome current challenges and fully realize the potential of photothermal therapy in clinical practice, offering hope for improved outcomes and quality of life for patients with oral cancer.

## 5. Relations between Metformin and Oral Cancer

Research has demonstrated an association between metformin use in diabetic patients and a reduced risk of developing oral cancer [86]. This inverse correlation has been observed across both genders, suggesting that metformin may offer a protective effect against oral cancer [87]. In a study conducted on Taiwanese patients with T2DM, metformin use was significantly associated with a lower incidence of oral cancer, particularly when the medication was used for more than 21.5 months [87]. The dose–response relationship indicated that longer durations and higher doses of metformin corresponded to a greater reduction in oral cancer risk. This protective effect was found to be independent of other anti-diabetic drugs and medications that might influence cancer risk, such as ACE inhibitors, angiotensin receptor blockers, statins, aspirin, and non-steroidal anti-inflammatory drugs. Although the concurrent use of other anti-diabetic drugs slightly weakened the protective effect of metformin, it did not entirely negate its benefits. These findings suggest that metformin could play a significant role in reducing the risk of oral cancer in patients with T2DM.

Metformin also shows promise in reducing the risk of HNSCC [88]. Clinical studies have found a significant association between metformin use and a lower incidence of HNSCC. Patients using metformin tend to experience reduced rates of loco-regional recurrence and metastasis, along with improved overall survival and disease-free survival rates, compared with those who do not use metformin [88]. Metformin’s potential benefits in this context include its ability to inhibit the growth of HNSCC cells and decrease mTORC1 activity, possibly preventing the development of HNSCC. The clinical evidence suggests that metformin contributes to improving overall survival outcomes in patients with HNSCC [88].

These findings emphasize the potential of metformin as a therapeutic agent beyond its primary use in diabetes management. Its possible role in cancer prevention and treatment, particularly for oral cancer and HNSCC, warrants further exploration [89]. Clinical data, particularly from Taiwanese patients with T2DM, highlights the importance of metformin not only as a glucose-lowering agent but also as a potential anticancer medication. Further research is needed to fully understand the mechanisms underlying metformin’s anticancer effects and to explore its potential applications in cancer prevention and treatment strategies. These clinical implications could lead to new therapeutic approaches that incorporate metformin in managing cancer risk among diabetic patients and potentially in the broader population.

## 6. How Metformin Inhibits Oral Squamous Cell Carcinoma

Metformin has exhibited multifaceted effects on OSCC cells, influencing various cellular pathways to inhibit cancer progression [86]. One key mechanism by which metformin acts is by inhibiting OSCC cell proliferation [90]. Metformin interferes with the proteolysis of the nerve growth factor receptor (NGFR), reducing the generation of its intracellular domain, NGFR-N [91]. Notably, NGFR-N has a strong affinity for the tumor suppressor protein p53 and inhibits its activity, thereby promoting cell proliferation [92]. However, in metformin-treated cells that overexpress NGFR, there is significant upregulation of p53-specific downstream transcripts and proteins, suggesting that metformin reverses the suppressive effect of NGFR-N on p53, thereby inhibiting OSCC cell proliferation [91].

Preclinical studies have also shown that metformin reverses the epithelial-to-mesenchymal transition (EMT) induced by CoCl2 in OSCC cells [93]. EMT is a critical process in cancer metastasis, involving changes that enable epithelial cells to acquire mesenchymal, invasive properties [94]. Metformin suppresses key signaling pathways that regulate EMT, including mTOR, HIF-1α, PKM2, and STAT3 [93]. By inhibiting these pathways, metformin reduces cell proliferation, migration, and invasion, thereby mitigating the metastatic potential of OSCC cells. The efficacy of metformin in OSCC has been demonstrated in both in vitro OSCC cell models and in vivo xenograft nude-mice models (Table 1). These preclinical studies suggest that metformin could offer a novel therapeutic strategy for treating OSCC by targeting multiple pathways involved in tumor progression and metastasis. Further preclinical research is needed to elucidate the precise mechanisms underlying metformin’s anticancer effects in OSCC and to confirm these findings before considering clinical applications in human patients [93].

Additionally, metformin has shown significant inhibitory effects on OSCC cell proliferation by inducing G1 phase cell cycle arrest in preclinical models [95]. It suppresses the migratory and invasive capacities of OSCC cells, promotes apoptosis, and induces autophagy, highlighting its multifaceted role in cancer cell regulation [95]. Interestingly, blocking autophagy has been found to enhance metformin’s efficacy against OSCC, suggesting that combining metformin with the autophagy inhibitor HCQ could be a promising therapeutic strategy for OSCC. This finding warrants further investigation in preclinical settings [95].

The LIN28/let-7 axis, crucial in OSCC, influences tumor development, progression, and prognosis [96]. LIN28 is an oncogenic protein that inhibits the maturation of the tumor suppressor microRNA let-7, promoting OSCC progression [96]. C1632, a small molecule inhibitor targeting LIN28, restores the tumor-suppressive function of let-7 [97]. Metformin indirectly influences the LIN28/let-7 axis through the activation of the AMPK pathway [98]. Together, C1632 and metformin synergistically combat OSCC by reducing cell proliferation, migration, and self-renewal abilities of OSCC cells in both in vitro and in vivo preclinical models [99]. This combined therapeutic approach leverages their complementary mechanisms, making it a promising strategy for treating OSCC. However, further preclinical validation is needed to confirm these findings before considering clinical translation.

In the clinical context, metformin use has been associated with a reduced risk of OSCC recurrence in patients with T2DM [100]. Studies have shown that T2DM patients with OSCC who use metformin exhibit significantly lower expression of EGFR compared with non-users [100]. Additionally, fluctuations in fasting blood glucose (FBG) levels have been positively correlated with EGFR expression in OSCC tissues of T2DM patients not on metformin therapy [101]. In recurrent OSCC, modulation of EGFR expression is critical for therapeutic outcomes. Metformin, known for its glucose-lowering effects, can exert antitumoral effects by targeting key oncogenic pathways, including EGFR [102,103]. Furthermore, combining metformin with 4SC-202 has been found to synergistically inhibit cancer cell growth and induce apoptosis by increasing ΔNp63 ubiquitination and degradation, both in vitro and in vivo [104]. This combination therapy presents a promising strategy for OSCC treatment, enhancing anticancer efficacy. Further clinical studies are necessary to validate and optimize these findings for potential clinical applications in OSCC therapy [104].

In summary, metformin shows significant potential as a therapeutic agent in OSCC treatment, with its ability to inhibit cancer cell proliferation, reverse EMT, induce apoptosis, and modulate key oncogenic pathways [5,10,90]. The observed synergistic effects when combining metformin with other therapeutic agents underscore its potential in comprehensive cancer treatment strategies. Continued research and clinical trials are essential to realize and fully optimize metformin’s application in OSCC treatment, providing new hope for improved patient outcomes in this challenging cancer type.

**Table 1:** Summary of studies investigating the therapeutic potential of metformin in oral cancer.

Study	Study Design	Sample Size	Key Findings	Limitations
Mekala et al. [11]	Retrospective Study	500 oral cancer patients, 500 controls	Metformin use is associated with a lower risk of oral cancer in diabetic patients	Potential for selection bias due to retrospective nature
Hammad et al. [44]	Review Article	Multiple studies reviewed	Explores metformin’s applications in dentistry, particularly its potential to prevent oral cancer	Dependent on the quality and consistency of reviewed studies
Wei et al. [91]	In vitro Study	OSCC cell lines	Metformin inhibits OSCC cell proliferation by interfering with NGFR-N and p53 pathways	In vitro findings need further validation in clinical settings
Yin et al. [93]	Experimental Study	OSCC cell lines	Metformin inhibits EMT in OSCC via the mTOR/HIF-1α/PKM2/STAT3 pathway	Study limited to cell lines; clinical relevance requires more studies
Chen et al. [99]	Experimental Study	In vitro and in vivo	LIN28 inhibitor combined with metformin reduces OSCC cell proliferation and migration	Preclinical study; human trials needed for confirmation
Tseng [87]	Cohort Study	Large cohort of diabetic patients	Metformin significantly reduces the risk of oral cancer in patients with T2DM	Observational study design limits causal inferences
Hu et al. [100]	Cohort Study	T2DM patients with OSCC	Metformin lowers EGFR expression and reduces the risk of OSCC recurrence	Observational nature limits the ability to establish causality
Gupta et al. [105]	Case-Control Study	Oral cancer patients	Elevated vitamin B12 levels were observed in oral cancer patients; metformin might affect this interaction	Small sample size and observational design limit generalizability
Broadfield et al. [16]	Experimental Study	In vivo and in vitro	Metformin modulates gut microbiome diversity, reducing tumor growth in colorectal cancer models	The direct applicability to oral cancer needs further exploration
He et al. [104]	In vitro and in vivo	OSCC models	Metformin combined with 4SC-202 promotes apoptosis in OSCC cells	Preclinical study; further research needed to confirm in human trials

## 7. The Challenge of Metformin in Oral Cancer

Metformin may encounter several significant challenges regarding drug interactions when considered for the prevention or treatment of oral cancer. One notable issue involves vitamin B12. A study in 2023 reported that vitamin B12 levels significantly increase among oral cancer patients [105]. Vitamin B12 is crucial for its antioxidant properties and its essential role in maintaining normal red blood cell development and proper nerve system function. This vitamin is naturally found in foods such as milk, meat, and eggs. However, it has been documented that patients taking metformin may experience a deficiency in vitamin B12 [106,107]. Insufficient vitamin B12 can lead to various oral diseases, including periodontal disease, due to the loss of antioxidants that help control ROS [108].

Additionally, metformin poses another challenge because of its potentially severe side effects, such as lactic acidosis. Severe lactic acidosis, particularly when accompanied by euglycemic diabetic ketoacidosis, can occur with an overdose of metformin. This side effect presents a significant risk in the treatment of oral cancer, as the appropriate dosage of metformin for this specific use is still undetermined, raising concerns about the possibility of overdosing [109,110].

The use of metformin in oral cancer therapy is further complicated by the need to balance its therapeutic benefits with the risks of side effects and drug interactions. While metformin’s role in cancer therapy is promising, given its potential to inhibit cancer cell proliferation and induce apoptosis, its interaction with essential nutrients such as vitamin B12 and the risk of severe metabolic disturbances require careful consideration and further research. The potential for metformin to cause vitamin B12 deficiency is particularly concerning, as this deficiency can weaken the overall antioxidant defense system and contribute to the development of additional oral health issues. Furthermore, determining the appropriate dosing regimen for metformin in the context of oral cancer treatment is a substantial challenge. The risk of overdosing, coupled with the potential for metformin to cause lactic acidosis, highlights the need for precise dosing guidelines and monitoring protocols to ensure patient safety while maximizing the therapeutic benefits of metformin.

In conclusion, while metformin shows significant potential as a therapeutic agent in the prevention and treatment of oral cancer, several formidable challenges must be addressed before it can be considered for formal clinical use. These challenges include managing drug interactions, particularly with vitamin B12, mitigating the risk of severe side effects such as lactic acidosis, and establishing safe and effective dosing guidelines. Extensive research and clinical trials are necessary to overcome these obstacles and fully realize the potential of metformin in oral cancer therapy. The journey towards integrating metformin into clinical practice for oral cancer treatment is complex and requires a thorough understanding of its pharmacological interactions, side effects, and therapeutic windows. Therefore, a cautious and well-researched approach is essential to ensure the safe and effective use of metformin in this new therapeutic domain.

## 8. Risk of Bias Assessment

This review carefully evaluated the risk of bias for each included study to provide a comprehensive understanding of the limitations and strengths of the current evidence base. A significant concern across many of the studies is the risk of selection bias, particularly in those employing retrospective designs. For instance, studies such as Mekala et al. (2020) [11] utilized retrospective data, which inherently introduces a high risk of selection bias due to the non-randomized selection of participants and the potential exclusion of relevant cases with incomplete records. This type of bias can skew results and affect the generalizability of the findings, as the selected sample may not accurately represent the broader population.

Additionally, information bias presents another critical issue, especially in studies where data collection relies heavily on self-reported measures or where diagnostic criteria are not consistently applied. For example, Gupta et al. (2023) [105] relied on self-reported data for metformin usage, which may lead to recall bias, thereby compromising the accuracy of the findings. Tseng (2016) [87] also demonstrated a risk of misclassification bias due to the lack of standardized outcome definitions, which could lead to inconsistencies in how outcomes are measured and reported.

Moreover, several studies exhibited reporting bias, where only significant results were emphasized while non-significant outcomes were either downplayed or omitted. This was notably observed in a study conducted by Hammad et al. (2023) [44], which selectively reported favorable outcomes without adequately discussing null results or potential limitations, potentially providing a skewed perception of metformin’s efficacy. The variability in study designs, such as differences in sample sizes, patient demographics, and treatment protocols, further contributes to the overall risk of bias. Studies such as that of Broadfield et al. (2022) [16] focused on colorectal cancer models, highlighting findings that may not be directly applicable to oral cancer, thereby introducing relevance bias and limiting the generalizability of their conclusions to the oral cancer context.

To address these concerns, we conducted a critical appraisal of each study’s methodology, including an assessment of sample size adequacy, study design robustness, and data collection methods. This rigorous evaluation aimed to identify potential biases and assess their impact on the validity and reliability of the study findings. Despite these efforts, the inherent biases present in many studies cannot be eliminated entirely and must be acknowledged as a limitation in interpreting the overall results of this review. It is crucial for future research to implement more rigorous study designs, such as prospective randomized controlled trials, to minimize these biases and provide more definitive evidence on the role of metformin in oral cancer therapy. The detailed risk of bias for each study included in this review is summarized in Table 2, which outlines the specific types of bias identified and their potential impact on the findings. This comprehensive assessment underscores the importance of considering these biases when interpreting the results and highlights the need for continued high-quality research in this area.

## 9. Conclusions

Metformin, a well-established antidiabetic drug, has shown promising potential in the treatment and prevention of oral cancer, particularly OSCC. Its multifaceted mechanisms of action, including the inhibition of cell proliferation, induction of apoptosis, and modulation of key oncogenic pathways, make it a valuable candidate for cancer therapy. Furthermore, metformin’s ability to reverse EMT and influence the LIN28/let-7 axis further underscores its comprehensive anticancer properties. However, it is crucial to address the challenges associated with its use, such as drug interactions, particularly with vitamin B12, and the risk of severe side effects such as lactic acidosis.

In this narrative review, we have critically evaluated each study’s methodology and results, considering factors such as sample size, study design, and the robustness of the conclusions drawn. While we included a broad range of studies to provide a comprehensive overview, the conclusions drawn from this review should be interpreted with caution, especially where data are limited or conflicting. Despite these limitations, the synergistic effects observed when metformin is combined with other therapeutic agents suggest its significant potential in integrated cancer treatment strategies. Continued research and clinical trials are essential to optimize metformin’s application in OSCC therapy, ensuring patient safety and maximizing therapeutic efficacy (Table 3). The exploration of metformin’s role in oral cancer management opens new avenues for improving patient outcomes and represents a significant advancement in the field of oncology.

## Figures and Tables

**Table 2:** Detailed risk of bias assessment.

Study	Study Design	Selection Bias	Information Bias	Reporting Bias	Other Biases	Overall Risk of Bias
Mekala et al. [11]	Retrospective Study	High	Moderate	Low	N/A	High
Hammad et al. [44]	Review Article	Low	High	High	Dependent on included studies	High
Wei et al. [91]	In vitro Study	N/A	Low	Moderate	Laboratory conditions	Moderate
Yin et al. [93]	Experimental Study	N/A	Moderate	Low	Limited to cell lines	Moderate
Chen et al. [99]	Experimental Study	N/A	Moderate	Low	Preclinical study design	Moderate
Tseng [87]	Cohort Study	Moderate	Low	Low	Observational design	Moderate
Hu et al. [100]	Cohort Study	Moderate	Low	Moderate	Observational design	Moderate
Gupta et al. [105]	Case-Control Study	High	Moderate	High	Small sample size	High
Broadfield et al. [16]	Experimental Study	N/A	Moderate	Low	Relevance to oral cancer	Moderate
He et al. [104]	In vitro and in vivo	N/A	Moderate	Low	Preclinical relevance	Moderate

**Table 3:** Summary of metformin applications, mechanisms, advantages, challenges, and research directions.

Item	Content
Applications of Metformin	Treatment of T2DM [19], oral cancer (especially OSCC) [11], cardiovascular and renal protection [23,31,33], PCOS [34], and other cancers (breast cancer [42], colorectal cancer [16], etc.)
Main Mechanisms of Action	- Inhibition of cell proliferation [91,95] - Induction of apoptosis [95,104] - Interference with the proteolysis of NGFR [91] - Reversal of EMT [93] - Inhibition of mTORC1 through AMPK pathway [24,44] - Influence on LIN28/let-7 axis [99]
Advantages	- Multiple mechanisms of action - Low cost [10] - Good tolerability [10] - Reduced cancer risk [6,8] - Improved patient outcomes [11,89] - Potential combination with other therapeutic agents [99,104]
Challenges	- Interaction with vitamin B12 [106,107] - Potential for severe side effects such as lactic acidosis [109,110] - Need to determine appropriate dosage [109,110] - Further research is needed to understand detailed mechanisms [6,11]
Clinical Studies and Evidence	- Clinical trials and observational studies show that metformin reduces the risk of oral cancer and improves treatment outcomes [11] - Animal models and cell experiments confirm its effects on inhibiting cancer cell proliferation and reversing EMT [91,93] - Enhanced therapeutic effects were observed when combined with other agents [99,104]
Future Research Directions	- Optimize the use of metformin - Explore synergistic effects with other treatments - Expand clinical trials to verify its efficacy and safety - Deepen understanding of metformin’s anticancer mechanisms and its potential applications in non-diabetic conditions

## Data Availability

The data presented in this study are available in this article.
